# Supplementary material for: Probing ultrafast spin-relaxation and precession dynamics in a cuprate Mott insulator with seven-femtosecond optical pulses
Source: Nat Commun. 2018 Sep 26;9:3948. doi: 10.1038/s41467-018-06312-z (PMC6158258; doi:10.1038/s41467-018-06312-z)
Supplement: Supplementary file 1 — Supplementary Information [file 41467_2018_6312_MOESM1_ESM.pdf]

Supplementary Information:

Probing ultrafast spin-relaxation and precession dynamics in a cuprate Mott insulator with seven-femtosecond optical pulses

T. Miyamoto et al.

Supplementary Note 1. Free induction decay signals and oscillatory components

The photoinduced reflectivity changes $\Delta R/R$ for $t < 0$ (Figs. 3a and 4a) are attributed to the free induction decay (FID) signals, which are explained as follows. A probe pulse generates a polarization, which emits an electromagnetic wave or equivalent light with the polarization frequency. This is observed as the reflected probe light. A pump pulse reaching the sample after the probe pulse modulates the probe-pulse-induced polarization, resulting in the modulation of the reflected probe light. With the decrease of $|t|$ at $t < 0$, such a modulation should be enhanced, which is consistent with the observations.

In the probe-energy dependence of $\Delta R/R$ (Fig. 4a), the oscillatory components appear not only at $t > 0$ but also at $t < 0$. By applying a Fourier filter to the time evolutions of $\Delta R/R$, we extracted the oscillatory components ($\Delta R_{\mathrm{OSC}}/R$) at $t < 0$ and $t > 0$, which are shown in Supplementary Figs. 1a and b, respectively. The oscillations in the FID signals were previously reported in organic materials and ascribed to coherent phonons coupled with the photoexcited state[1,2]. In Nd_2CuO_4, the spin system is strongly coupled with the photoexcited state. Consequently, the oscillatory component in the FID signals can also be assigned to the spin excitations or magnons. The Fourier power spectra of $\Delta R_{\mathrm{OSC}}/R$ are shown in Supplementary Fig. 1c. The peak energy of each oscillatory component at $t < 0$ (orange lines) is almost equal to that at the same probe photon energy (blue lines). Therefore, the oscillatory components at $t < 0$ and $t > 0$ have the same origin.

Supplementary Note 2. Fitting analyses of the time evolution of reflectivity changes

To analyze the time evolution of $\Delta R(t)/R$, we adopted the fitting function

(Supplementary Equation 1), which is given as

$$\frac{\Delta R(t)}{R} = -\left\{A_1 + A_2\left[1 - \exp\left(-\frac{t}{\tau_{\rm r}}\right)\right]\right\}\exp\left(-\frac{t}{\tau_1}\right) - A_3\exp\left(-\frac{t}{\tau_2}\right) \qquad (1)$$

The first term on the right-hand side represents the mid-gap-absorption component ascribed to magnetic polarons. The second term, $-A_3\exp\left(-\frac{t}{\tau_2}\right)$, shows the formation and decay of the Drude component, which is neglected in Equation 1. The main text describes the physical meanings of the parameters. In the fitting procedures, each term in Supplementary Equation 1 or Equation 1 is convolved with the Gaussian profile corresponding to the time resolution (10 fs). From the values of A_3 and τ_2, the excitation photon density dependence of the magnitude and the decay time of the Drude component can be discussed. For a weak excitation density, $x_{\rm ph}$ = 0.0016, 0.0032, and 0.0049 ph/Cu, the Drude component can be neglected.

Supplementary Figure 2a-l replots all of the time evolutions of the reflectivity changes displayed in Fig. 3a by green open circles, which are well reproduced by the fitting curves (thin black lines) except for $t < 0$. The time evolutions of the first and second terms are represented in Supplementary Fig. 2m-x by blue and red lines, respectively. The values of parameters A_3 and τ_2 are shown in Figs. 3f and g, respectively.

Supplementary Note 3. Time evolutions of the photoinduced reflectivity changes in the long-time domain

In Fig. 2 and Supplementary Fig. 2, we analyzed the time evolutions of the photoinduced reflectivity changes $\Delta R/R$ from $t = -100$ fs to $t = 200$ fs to obtain the fitting parameters. To evaluate more precisely the recombination time of carriers

(magnetic polarons), τ_1, we measured $\Delta R/R$ in the long-time region up to 500 fs. Supplementary Figure 3 shows the results for both the weak excitation ($x_{\mathrm{ph}} = 0.0049\ \mathrm{ph/Cu}$) and strong excitation ($x_{\mathrm{ph}} = 0.079\ \mathrm{ph/Cu}$). The data in the short-time region are the same as those presented in Supplementary Figs. 2c and l. In $\Delta R/R$ at $t > 100\ \mathrm{fs}$, a component with a long decay time exists. This component may be attributed to heating of the system, which originates from the recombination of carriers[3,4]. To include this thermal-effect component in the analyses, the third term is added to Supplementary Equation 1.

$$\frac{\Delta R(t)}{R} = -\left\{A_1 + A_2\left[1 - \exp\left(-\frac{t}{\tau_{\mathrm{r}}}\right)\right]\right\}\exp\left(-\frac{t}{\tau_1}\right) - A_3\exp\left(-\frac{t}{\tau_2}\right) - A_4\left[1 - \exp\left(-\frac{t}{\tau_1}\right)\right] \quad (2)$$

It is reasonable to consider that the rise time of the thermal-effect component is the same as the decay time of carriers τ_1[4]. In the fitting procedures, we used the same parameter values of A_2/A_1, τ_{r}, and τ_2 as those used in Supplementary Figs. 2c(o) and l(x). The black lines in Supplementary Fig. 3 show the fitting curves. The decay dynamics in the long-time region can be well reproduced with $\tau_1 = 350$ fs.

Supplementary Note 4. Theoretical analyses of coherent oscillations on reflectivity changes

To explain the generation of the coherent oscillations in the pump-probe responses, we considered a simple semi-phenomenological model given by

$$H_0 = \hbar\omega_{\mathrm{CT}} X^{\dagger} X + \sum_k \hbar\omega_k b_k^{\dagger} b_k\ , \quad (3)$$

where $X^\dagger(X)$ is the creation (annihilation) operators for a doublon-holon pair with an energy of $\hbar\omega_{\mathrm{CT}}$ and $b_k^\dagger(b_k)$ is a magnon with the momentum k and the energy of $\hbar\omega_k$. The electronic and spin states are represented by $|N, n\rangle$, where N (=1 or 2) denotes the number of doublon-holon pairs and n denotes a two-magnon excited state with a frequency of $2\hbar\omega_n$, respectively. For simplicity, a photon with the frequency ω is assumed to excite specifically a doublon-holon pair and two-magnons with the energy of $2\hbar\omega_k$, which is expressed by the following Hamiltonian

$$H' = A_{\omega_{\mathrm{CT}}} X^\dagger + g \sum_k A_{\omega_{\mathrm{CT}}+2\omega_k} X^\dagger b_k^\dagger b_{-k}^\dagger + H.c. \qquad (4)$$

A_ω is the component of the vector potential with frequency ω, and g is the coupling constant. A pump pulse introduced at $t = 0$ generates a photoexcited state $|\Psi(0)\rangle$, which is represented by a linear combination of the initial state $|0\rangle$, one doublon-holon pair state $|1,0\rangle$, and the two-magnon sideband state $|1, n\rangle$. The electronic current coupled with the vector potential is identified from H' in Supplementary Equation 4. The expectation of the electronic current j at t, $\langle j\rangle(t)$, is calculated as $\langle j\rangle(t) = \langle\Psi(t)|j|\Psi(t)\rangle$ using the time-dependent wave function $|\Psi(t)\rangle = e^{-iH_0 t}|\Psi(0)\rangle$.

Here we demonstrate that the coherent oscillation appears in the simplified model from Supplementary Equation 3, where the continuous magnon spectrum is replaced by two discrete energy levels labeled as K and Q. The corresponding two-magnon energies are given by $\hbar\Omega_K\ (= 2\hbar\omega_K)$ and $\hbar\Omega_Q\left(= 2\hbar\omega_Q > \hbar\Omega_K\right)$, respectively. The energy levels of the excited states considered in this model are shown in Supplementary Fig. 4a. The current expectation value is approximately given as $\langle j\rangle(t) \sim \int_0^t dt' F(t')$ with

$$
\begin{aligned}
F(t') = {} & w_1 \sin \omega_{\mathrm{CT}}(t-t')\, A'_{\omega_{\mathrm{CT}}}(t') \\
& + w_2 \sin[(\omega_{\mathrm{CT}}+\Omega_K)(t-t')]\, A'_{\omega_{\mathrm{CT}}+\Omega_K}(t') \\
& + w_3 \sin\left[\left(\omega_{\mathrm{CT}}+\Omega_Q\right)(t-t')\right] A'_{\omega_{\mathrm{CT}}+\Omega_Q}(t') \\
& + w_4 \{\sin[\omega_{\mathrm{CT}}t-(\omega_{\mathrm{CT}}+\Omega_K)t']\, A'_{\omega_{\mathrm{CT}}+\Omega_K}(t') \\
& \quad + \sin[(\omega_{\mathrm{CT}}+\Omega_K)t-\omega_{\mathrm{CT}}t']\, A'_{\omega_{\mathrm{CT}}}(t')\} \\
& + w_5 \left\{\sin\left[\omega_{\mathrm{CT}}t-\left(\omega_{\mathrm{CT}}+\Omega_Q\right)t'\right] A'_{\omega_{\mathrm{CT}}+\Omega_Q}(t') \right. \\
& \quad \left. + \sin\left[\left(\omega_{\mathrm{CT}}+\Omega_Q\right)t-\omega_{\mathrm{CT}}t'\right] A'_{\omega_{\mathrm{CT}}}(t')\right\} \\
& + w_6 \left\{\sin\left[(\omega_{\mathrm{CT}}+\Omega_K)t-\left(\omega_{\mathrm{CT}}+\Omega_Q\right)t'\right] A'_{\omega_{\mathrm{CT}}+\Omega_Q}(t') \right. \\
& \quad \left. + \sin\left[\left(\omega_{\mathrm{CT}}+\Omega_Q\right)t-(\omega_{\mathrm{CT}}+\Omega_K)t'\right] A'_{\omega_{\mathrm{CT}}+\Omega_K}(t')\right\}, \qquad (5)
\end{aligned}
$$

where w_l $(l = 1-6)$ is a constant and A'_ω is the vector potential for the probe photon. The 4th term (5th term) represents the interference between the processes of $|1,0\rangle \rightarrow |2,K\rangle$ and $|1,K\rangle \rightarrow |2,K\rangle$ ($|1,0\rangle \rightarrow |2,Q\rangle$ and $|1,Q\rangle \rightarrow |2,Q\rangle$), which is schematically shown in Supplementary Fig. 4b. When $t \sim t'$, these terms show a coherent oscillation with frequency Ω_K $\left(\Omega_Q\right)$. The last two terms represent the interferences between the processes of $|1,K\rangle \rightarrow |2,K,Q\rangle$ and $|1,Q\rangle \rightarrow |2,K,Q\rangle$. In an actual situation where two-magnon states are continuous, these terms do not give coherent oscillations.

To show a coherent oscillation due to these interferences, the probe vector potential is set to be a damped oscillator given as $A'_\omega(t) = f_\omega e^{-(t-t_0)^2/\tau^2} \cos\omega(t-t_0)$ with $f_\omega = e^{-(\omega-\omega_0)^2/\delta\omega^2}$. Supplementary Figures 4c and d show parts of $\langle j\rangle(t)$ where the 4th and 5th terms in Supplementary Equation 5 (i.e., the interference terms) are adopted in the calculations. Fourier transforms of Supplementary Figs. 4c and d are shown in Supplementary Figs. 4e and f, respectively. The frequencies of the probe photons are tuned at $\omega_0 = \omega_{\mathrm{CT}} + \Omega_K$ and $\omega_0 = \omega_{\mathrm{CT}} + \Omega_Q$ in Supplementary Figs. 4c and d, respectively. The parameter values are chosen as $\Omega_K = 1, \Omega_Q = 2, \omega_{\mathrm{CT}} = 10, \tau = 0.5$,

and $\delta\omega = 1$. The results show that the current spectra have a peak structure near Ω_K (Ω_Q), when the probe photons are tuned around $\omega_{\mathrm{CT}} + \Omega_K$ $(\omega_{\mathrm{CT}} + \Omega_Q)$, giving an explanation of the characteristic coherent oscillation experimentally observed.

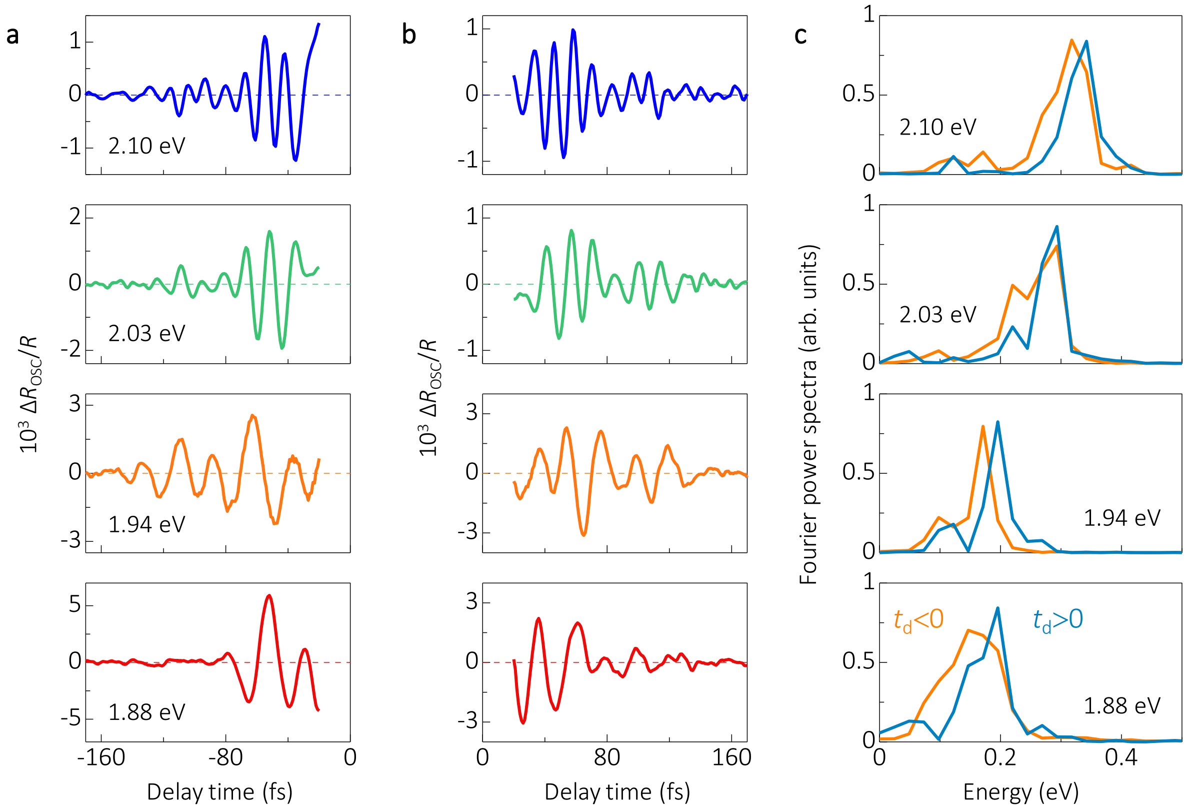

Supplementary Figure 1 | Probe energy dependence of the oscillations on $\Delta R/R$. a,b, Time evolutions of the oscillatory components $\Delta R_{\mathrm{OSC}}/R$ (**a**) at $t < 0$ and (**b**) at $t > 0$ extracted from the data shown in Fig. 3a. The probe energies are shown in **a**. **c,** Fourier power spectra of $\Delta R_{\mathrm{OSC}}/R$ at $t < 0$ shown in Fig. **a** (orange lines) and at $t > 0$ shown in Fig. **b** (blue lines).

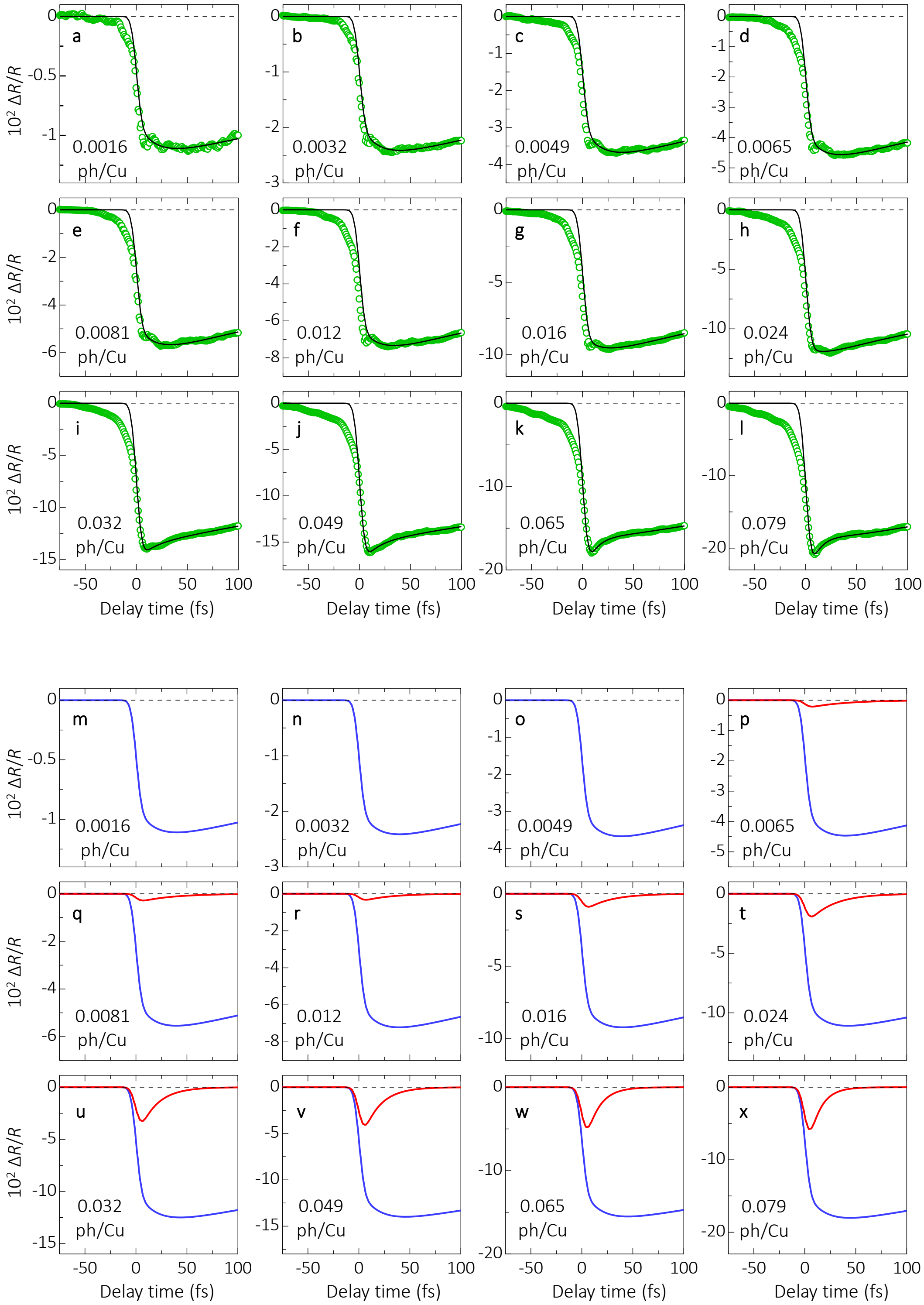

Supplementary Figure 2 | Fitting analyses of the time evolutions of $\Delta R/R$. a-l, Open circles and black lines show time evolutions of $\Delta R/R$ and the fitting curves for x_{ph} = 0.0016-0.079 ph/Cu. **m-x,** Blue and red lines represent the mid-gap-absorption components and the Drude components, respectively, which are obtained by the fitting analyses.

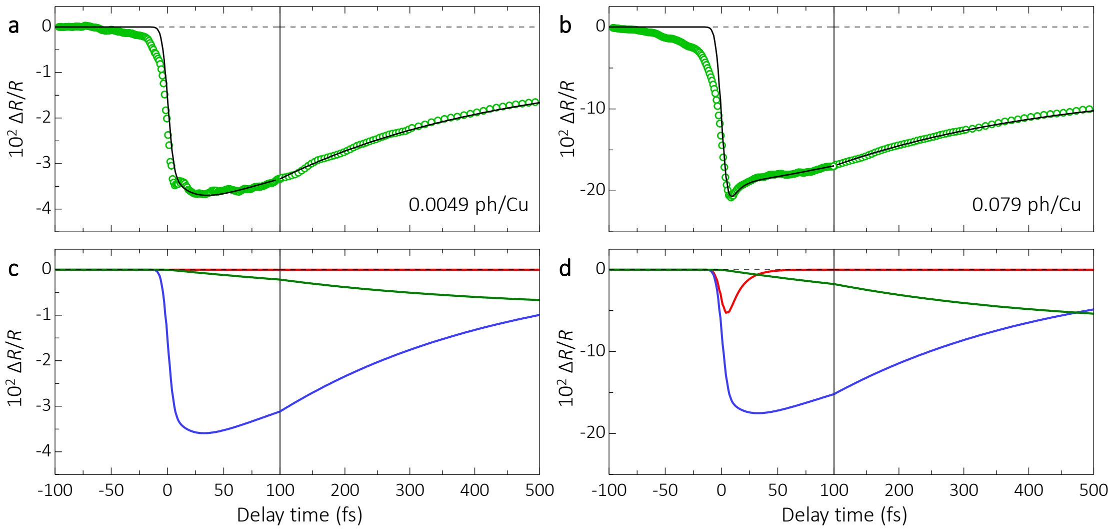

Supplementary Figure 3 | Analyses of Δ*R*/*R* in the long time region. a,b, Green open circles and black lines show the time evolutions of $\Delta R/R$ and the fitting curves for the weak excitation ($x_{\mathrm{ph}} = 0.0049\ \mathrm{ph/Cu}$) in (**a**) and the strong excitation ($x_{\mathrm{ph}} = 0.079\ \mathrm{ph/Cu}$) in (**b**). **c,d,** Blue, red, and green lines represent the mid-gap-absorption component, the Drude component, and the thermal-effect component, respectively, obtained by the fitting analyses.

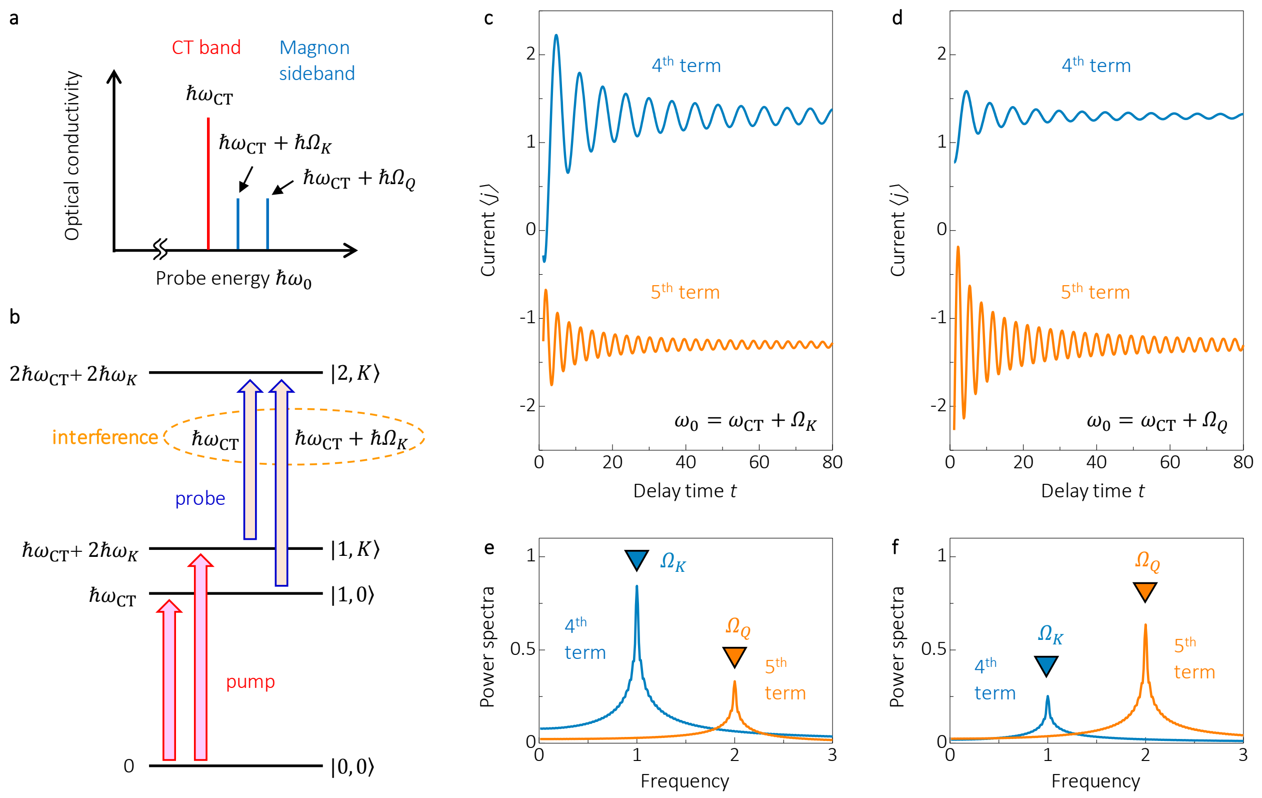

Supplementary Figure 4 | Time-dependent currents and Fourier power spectra calculated in a simplified system with two different frequencies of magnons. a, The energy levels assumed in the model. **b,** A schematic of the interference between the processes of $|1, 0\rangle \rightarrow |2, K\rangle$ and $|1, K\rangle \rightarrow |2, K\rangle$. **c,d,** Time dependent currents for the 4th term (blue lines) and the 5th term (orange lines) in Supplementary Equation 5. They are vertically offset for clarity. The frequencies of the probe pulse are set at around $\omega_{CT} + \Omega_K$ in (**c**), and $\omega_{CT} + \Omega_Q$ in (**d**). **e,f,** Fourier power spectra of (**c**) in (**e**) and (**d**) in (**f**).

Supplementary References

1. Kobayashi, T., Du, J., Feng, W. & Yoshino, K. Excited-state molecular vibration observed for a probe pulse preceding the pump pulse by real-time optical spectroscopy. *Phys. Rev. Lett.* **101**, 037402 (2008).

2. Matsubara, Y. *et al*. Coherent dynamics of photoinduced phase formation in a strongly correlated organic crystal. *Phys. Rev. B* **89**, 161102(R) (2014).

3. Okamoto, H. *et al*. Ultrafast charge dynamics in photoexcited Nd_2CuO_4 and La_2CuO_4 cuprate compounds investigated by femtosecond absorption spectroscopy. *Phys. Rev. B* **82**, 060513(R) (2010).

4. Okamoto, H. *et al*. Photoinduced transition from Mott insulator to metal in the undoped cuprates Nd_2CuO_4 and La_2CuO_4. *Phys. Rev. B* **83**, 125102 (2011).
